# Intranasal Administration of the Antisecretory Peptide AF-16 Reduces Edema and Improves Cognitive Function Following Diffuse Traumatic Brain Injury in the Rat

**DOI:** 10.3389/fneur.2017.00039

**Published:** 2017-02-14

**Authors:** Fredrik Clausen, Hans-Arne Hansson, Johan Raud, Niklas Marklund

**Affiliations:** ^1^Unit for Neurosurgery, Department of Neuroscience, Uppsala University, Uppsala, Sweden; ^2^Institute of Biomedicine, University of Gothenburg, Göteborg, Sweden; ^3^Lantmännen AS Faktor AB, Stockholm, Sweden

**Keywords:** cerebral edema, traumatic brain injury, intranasal, neuroprotection, AF-16, cognition

## Abstract

A synthetic peptide with antisecretory activity, antisecretory factor (AF)-16, improves injury-related deficits in water and ion transport and decreases intracranial pressure after experimental cold lesion injury and encephalitis although its role in traumatic brain injury (TBI) is unknown. AF-16 or an inactive reference peptide was administrated intranasally 30 min following midline fluid percussion injury (mFPI; *n* = 52), a model of diffuse mild-moderate TBI in rats. Sham-injured (*n* = 14) or naïve (*n* = 24) animals were used as controls. The rats survived for either 48 h or 15 days post-injury. At 48 h, the animals were tested in the Morris water maze (MWM) for memory function and their brains analyzed for cerebral edema. Here, mFPI-induced brain edema compared to sham or naïve controls that was significantly reduced by AF-16 treatment (*p* < 0.05) although MWM performance was not altered. In the 15-day survival groups, the MWM learning and memory abilities as well as histological changes were analyzed. AF-16-treated brain-injured animals shortened both MWM latency and swim path in the learning trials (*p* < 0.05) and improved probe trial performance compared to brain-injured controls treated with the inactive reference peptide. A modest decrease by AF-16 on TBI-induced changes in hippocampal glial acidic fibrillary protein (GFAP) staining (*p* = 0.11) was observed. AF-16 treatment did not alter any other immunohistochemical analyses (degenerating neurons, beta-amyloid precursor protein (β-APP), and Olig2). In conclusion, intranasal AF-16-attenuated brain edema and enhanced visuospatial learning and memory following diffuse TBI in the rat. Intranasal administration early post-injury of a promising neuroprotective substance offers a novel treatment approach for TBI.

## Introduction

Traumatic brain injury (TBI) is a global health problem caused by e.g. motor vehicle accidents, falls, assaults, as well as work and sports injuries (1–4). Though TBI has a broad severity spectrum, even mild and moderate injuries frequently leave the patient with notable and persistent deficits. The initial brain injury occurring at time of impact is markedly exacerbated over hours, days, and months by complex secondary cascades (5, 6). In theory and as reported in many preclinical studies, these secondary injury factors should be amenable to pharmacological interventions resulting in improved clinical outcome. However, despite extensive experimental research and many clinical trials, no pharmacological treatment has shown successful results in TBI patients (7). One of the most life-threatening secondary insults is cerebral edema, which can rapidly increase the intracranial pressure (ICP) to deleterious levels. The mechanisms behind the development of post-traumatic brain edema are complex, and both cytotoxic and vasogenic components contribute to the increase in brain water content (BWC) (7).

Diffuse axonal injury (DAI) was initially described in the 1950s in patients who died early following severe TBI (8). Later, in the early 1980s, it was described as a separate clinicopathological entity different from focal TBI in both the clinical setting (9) and in experimental models of acceleration injury TBI (10). A key pathophysiological event is the pathological deformation and stretching of axons after TBI resulting in dysfunctional axonal transport possibly leading to axonal transection. Injury to axons leads to brain network dysfunction, and the resulting DAI induces cognitive impairment and long-term sequelae (11, 12). Despite these severe clinical consequences, there is no beneficial pharmacological therapy available for DAI.

Antisecretory Factor (AF) is an endogenous protein with several pharmacological actions, most evidently preventing abnormal flow of fluid across cellular barriers and being anti-inflammatory. To date, the most evaluated effects being the protection against diarrheal disease and intestinal inflammation (13). However, AF has not been shown to influence fluid distribution in normal tissue, nor exert any toxicity under normal conditions (14–17). A 16-peptide fragment, named AF-16, was developed as a pharmacological compound for intranasal administration, showing positive results on brain edema formation in both cold lesion injury (15, 16) and experimental encephalitis, in the latter study significantly improving survival due to reduced ICP without influencing the inflammatory response (18). Using the intranasal route of administration, AF-16 was observed to enter the brain and was detected in the cerebrospinal fluid [CSF (16)]. In the present study, we used a clinically relevant DAI model, the midline fluid percussion brain injury (mFPI), in rats and hypothesized that intranasal AF-16 could attenuate the traumatically induced cerebral edema, behavioral impairment, and various TBI-induced histological factors. We choose a mild–moderate diffuse TBI model since this represents the most common injury type seen clinically. We focused our evaluation on the cognitive performance in the Morris Water Maze [MWM (19)]; in view of our experience that the commonly used tests of gross motor function are less sensitive in diffuse than in focal TBI models. Although systemic biomarkers may be important in prognosis, injury characterization and monitoring in clinical TBI (20, 21), this approach would require a much larger study using multiple time points and study groups. Instead, we focused our histological outcome on aspects of cell death, axonal and white matter injury, and astrogliosis known to be present in the mFPI model. Our results show that the brain edema was attenuated at 48 h and the visuospatial learning performance was enhanced at least 15 days post-injury without significantly influencing histological outcome measures. These results suggest that AF-16 is a promising neuroprotective treatment of TBI and that intranasal administration is an attractive alternative route for the delivery of potentially beneficial pharmacological compounds to the injured brain.

## Materials and Methods

### Animals

The animal experiments were approved by the Uppsala County Animal Ethics board and followed the rules and regulations of the Swedish Agricultural Board. All evaluations were performed by a researcher blinded to the treatment status of each animal.

Male Sprague-Dawley rats (350–400 g, *n* = 90) were used; 52 were subjected to midline fluid percussion injury (mFPI), 14 were used as sham-injured controls, and 24 rats were used as naïve controls. Forty animals were used for a 48-h survival time-point post-injury, and 50 animals were used for a 15-day survival study. The number of animals in each group and time point is shown in Table 1.

**Table 1:** **Number of animals in the different treatment groups**.

	Naïve + scramble	Naïve + antisecretory factor (AF)-16	Sham + scramble	mFPI + scramble	mFPI + AF-16
48-h survival	6	6	6	8	9
15-day survival	6	6	8	14	13

### Animal Surgery

Following induction of anesthesia with 4.5% isoflurane in air for 3 min, the animals were moved to a stereotaxic frame and kept anesthetized using a nose cone with a mixture of nitrous oxide/oxygen (70/30%) and isoflurane (1.4%). Core body temperature was maintained at 37 ± 0.5°C using a rectal thermometer coupled to an automatically regulated heating pad (CMA150, CMA, Stockholm, Sweden). The scalp was opened by a midline incision after application of bupivacaine. The mFPI brain injury model was used according to previously used protocols (22, 23). In brief, a 4-mm circular craniotomy was performed over the midline 2 mm behind the bregma suture. An anchoring screw was attached to the skull bone, the trauma cap attached to the bone using tissue adhesive (Histoacrylic, Braun Aesculap AG, Tuttlingen, Germany), and dental cement (Dentalon; Hureaeus Kulzer, Hanu, Germany) was poured over the skull and allowed to set. The animal was moved to the FPI-device, the piston was released, and TBI was induced, delivering a pressure of 2.20 ± 0.12 atm. The duration of apnea was recorded and the animal was allowed to stabilize its breathing pattern before being reattached to the stereotaxic frame. The trauma cap and dental cement was removed, the bone piece replaced and secured with tissue adhesive. The scalp was sutured and the animal allowed to recover in a heated cage. Sham-injured controls were subjected to the entire surgical procedure except that the piston was not released. At 48 h or 15 days post-mFPI or sham injury, the rats were over-anesthetized using an i.p. injection of sodium pentobarbital (0.5 ml of Euthasol^®^, 400 mg/l pentobarbital), and the brains removed for determination of BWC or tissue analysis (see below).

### Treatment

To balance for individual cognitive performance, we divided the animals according to ability to learn to locate the hidden platform in the MWM prior to the injury into the different groups with similar MWM learning latencies (the short-term study). For both studies, AF16 or Scrambled peptides were prepared in vials labeled A or B by a researcher not involved in the study and unaware of the study design, where the code was not revealed until the completion of the study. In the long-term study, to balance the treatments groups of brain-injured animals in terms of injury severity, we used the apnea time to create groups with similar apnea times. The naïve animals were randomly assigned to either treatment A or B.

The treatment peptide AF16 or the scrambled inactive peptide (hereafter named Scramble) were instilled intranasally by briefly sedating the rats with isoflurane (4% in air for induction and 1% in air through a nosecone for maintaining sedation) and gently injecting 25 µl per nostril of isotonic saline solution with scramble or AF-16 peptide (2 mg/ml), as previously described (15, 16). The first treatment was administered 30 min after mFPI or sham injury and at a corresponding time point for naïve animals. At 6 h after the first administration of AF-16 or scramble peptide, an additional dose was given and then twice the following day for the 48-h survival animals and twice daily for the following 3 days for the animals with 15 days survival after surgery. Several previous studies showed that the scramble peptide is inert, and also that AF-16 is without adverse events in naïve animals (15, 24). In addition, no influence on ICP nor other physiological parameters were observed in sham-exposed animals (15, 24). Since animal ethics regulation requires that animal number should be reduced whenever possible and without compromising scientific value, we, therefore, chose to omit an AF-16 -treated, sham-injured group since the study design also included an AF-16-treated naïve group.

### Brain Water Content

Immediately following sacrifice, the brain was rapidly removed from the cranium and cut into three coronal tissue samples of equal size. The most anterior sample was frontal to the impact/craniotomy site and was discarded from further analysis. Each tissue sample was placed on pre-weighed sheets of aluminum foil that was weighed immediately following removal to obtain the wet weight (WW). Each tissue sample was then dried in a pre-heated oven at 80°C for 72 h and weighed once more to obtain dry weight (DW). Water content was calculated as a percentage of WW according to the formula % brain water content (BWC) = (WW − DW)/WW × 100.

### Morris Water Maze

Using the MWM paradigm as previously described (25, 26), spatial learning and memory was evaluated. The MWM consists of a 1.4-m diameter circular tank and a 10-cm diameter white platform, placed in the southwest quadrant of the tank and submerged 1 cm below the surface of the 28°C water. Simple visual cues to aid navigation were placed on roller curtains surrounding the pool.

Each swim trial was run by placing the rat in the tank at one of four designated entry points (W, N, E, and S) facing the wall, activating the video-based computer tracking system (HVS Image Ltd., Buckingham, UK), and the trial was terminated when the rat located the platform. The rat was allowed to remain undisturbed on the platform for 15 s to acquire the visual cues surrounding the pool. If the animal failed to locate the platform within 120 s, it was placed on the platform for 15 s. Each learning trial was analyzed for latency to locate the hidden platform, distance swum, and swim speed.

The animals with 48 h survival time were trained prior to sham injury or mFPI using four learning trials per day with 30 min rest interval between trials for 2 days before TBI and subjected to the memory test (probe trial) at 48 h post-injury. The rats with 15 days survival were evaluated for learning ability in the MWM using four learning trials per day for 4 days (day 9 to 12 after TBI) followed by the memory (probe) trial test at day 15 after TBI. In the memory (probe) trial test, the platform was removed and the trial was analyzed for latency to find the platform site, number of crosses over the platform site, and time spent in the correct quadrant of the pool.

### Hemispheric and Ventricle Volumes

Rats sacrificed 15 days after mFPI or sham injury were transcardially perfused with isotonic saline followed by phosphate-buffered 4% formaldehyde (Histolab AB, Gothenburg, Sweden). Following rapid removal, the brain was placed in 4% formaldehyde in phosphate-buffered saline (PBS) at 4°C for 24 h and then in 30% w/v sucrose at 4°C for 72 h. It was then snap-frozen in isopentane (Sigma, Stockholm, Sweden) at -55°C. Sections were made in a cryostat (HM500; Microm, Walldorf, Germany) at −22°C.

Nine coronal sections from bregma levels 0 to −5 mm, approximately 0.5 mm apart, were stained with Mayer’s Hematoxylin and Eosin (Histolab) and, using a digital camera (Olympus), photographed in a stereomicroscope (Zeiss Stemi 2000-C; Zeiss Gmbh, Göttingen, Germany). The hemispherical volume and cortical lesion volume were calculated using the Sectiontovolume software according to a previously published protocol (27).

### Immunohistochemistry

Four primary antibodies (Table 2) were used to characterize the brain injury morphologically and compare it to the brains of sham-injured and naïve controls. Frozen sections on SuperFrost+ slides were thawed for 5 min before being placed into chilled acetone for 1 min of post-fixation. The sections were washed for 5 min in 0.1 M PBS. Endogenous peroxidation was blocked using the solution provided in the MACH-1 kit (Biocare Medical, Concord, CA, USA) for 5 min. The sections were then washed for 5 min in PBS. Cross-reactive protein binding was blocked using the solution provided in the MACH-1 kit for 15 min. Excess protein block was tapped off, the primary antibody was applied to the sections and incubated overnight in 4°C. The sections were then washed three times in PBS before the probe provided in the MACH-1 kit was applied and incubated for 15 min. The sections were washed three times for 5 min in PBS. The HRP polymer provided in the MACH-1 kit was applied and incubated for 30 min. The sections were washed three times for 5 min in PBS. As chromogen, the diamino-benzidine (DAB) solution supplied in the MACH-1 kit was used and applied for 5 min before rinsing in deionized water for 5 min. The sections were counterstained by a brief dip in Mayer’s hematoxylin (Histolab, Gothenburg, Sweden), followed by rinsing in deionized water for 5 min. The sections were dried in an alcohol to xylen ladder before coverslips were mounted using Pertex (Histolab). In negative controls, the same protocol was run except that the primary antibody was omitted.

**Table 2:** **Primary antibodies used for immunohistochemical evaluation**.

Antibody	Target	Manufacturer	Dilution used in experiments
MAP2 (M9942)	Neuron (somas and dendrites)	Sigma	1:500
GFAP (M0761)	Reactive astrocytes	Dako	1:200
Beta-APP (51-2700)	Axonal deposits	Life technologies	1:200
Olig2 (AB9610)	Mature and immature oligodendrocytes	MerckMillipore	1:500

Different regions of interest (ROI) from four stained sections from bregma −1.0 to −3.0 mm were photographed at 100× magnification using light microscopy (Zeiss Axiovision, Zeiss Gmbh) resulting in micrographs in the size of 1.39 mm × 1.04 mm. The micrographs were analyzed using the Zen software (Zeiss Gmbh) to measure the area positively stained for GFAP in the hilus of the dentate gyrus (HDG) and CA3 of the hippocampus. The thresholds for positive staining was manually set by defining a region positive for GFAP, and this threshold was then automatically applied by the software on all sections throughout the analysis. The CA3 area with loss of MAP2 staining was manually outlined for each section, where only an area with clearly reduced staining compared to neighboring, intact tissue was included in the analysis. The number of MAP2-positive cells with pyknotic cell nuclei in the CA3 region was manually and exhaustively counted. For the β-APP and Olig2 analyses, the ROIs were in the white matter tracts of the corpus callosum, external capsule, cingulate gyrus and the striatum, and the number of positive deposits or cells, respectively, were manually and exhaustively counted. The average number per ROI was statistically analyzed.

### Statistics

All data were sampled and entered at time of the experiments whenever possible and handled in Microsoft Excel (Microsoft, Redmond, WA, USA) before it was evaluated using Statistica (Statsoft, Tulsa, OK, USA). The data for the different experiments were tested for normal distribution using Shapiro–Wilk’s test. If the data were normally distributed, a one-tailed two way ANOVA for injury and treatment was performed. If the data were not normally distributed, a non-parametric one way and one-tailed ANOVA using Kruskal–Wallis test was used and followed by Mann–Whitney tests between parameters. A *p*-value of below 0.05 was considered significant. Parametric statistics were used for weight, lesion volume, MWM, and BWC analyses while non-parametric tests were used for the immunohistochemistry analyses.

## Results

### Surgery

**Figure 1:**
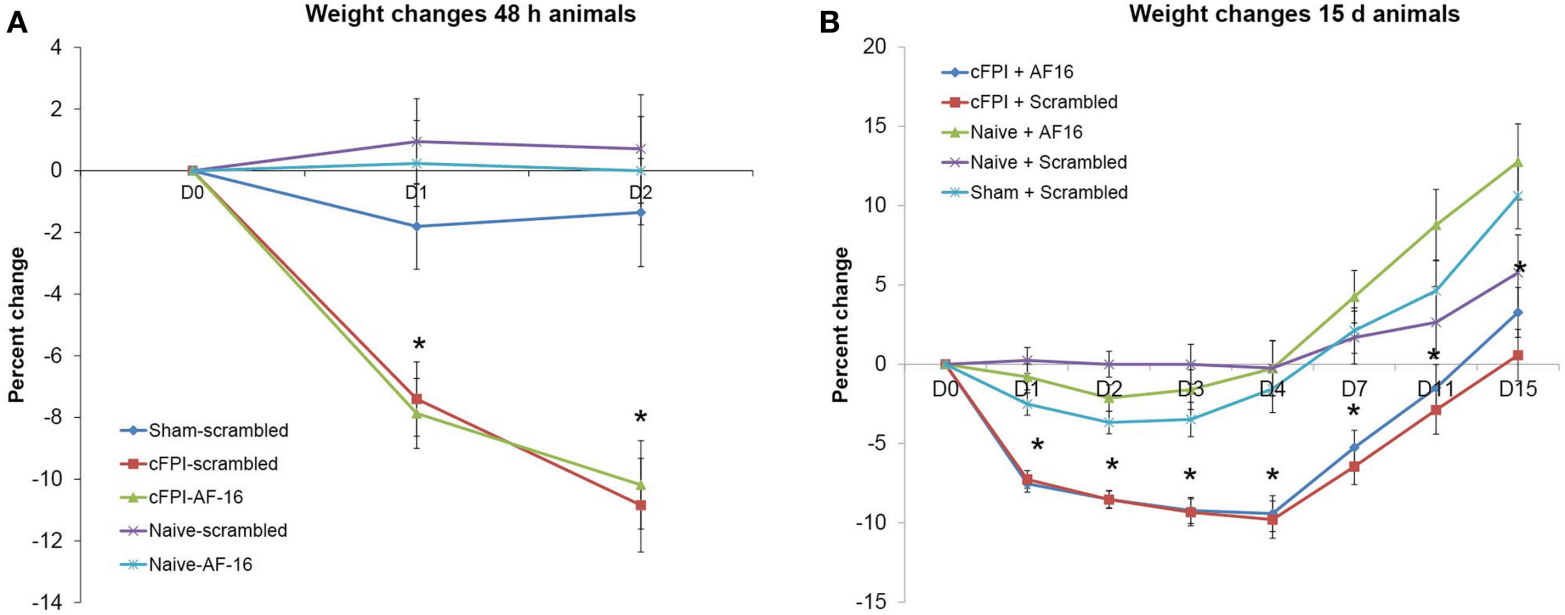
**Post-injury weight changes (means ± SEM)**. **(A)** Weight changes in the 48-h survival study. A significant weight loss in brain-injured animals was observed compared to sham-injured and naive animals (indicated with an asterisk, *p* < 0.05) at post-injury day 1 and 2. Treatment with antisecretory factor (AF)-16 or the inactive control (scramble) did not influence post-injury weight loss. **(B)** Weight changes in the long-term study. A persistent weight loss in brain-injured animals compared to sham-injured and naive controls were observed (indicated with an asterisk, *p* < 0.05) that was not influenced by AF-16 or the inactive control (scramble) treatment.

In the short-term study with 48 h post-injury survival time, three out of 20 animals died in the immediate post-operative period resulting in a 15% acute mortality. The mean post-traumatic apnea was 38.6 ± 4.6 s for the animals treated with the inactive, scrambled peptide and 40.0 ± 2.9 s for AF16-treated animals. A significant weight loss was induced by the brain injury when compared to sham-injured controls (*p* < 0.05) although it was not influenced by the treatment compound (Figure 1A).

In the long-term study with 15 days post-injury survival time, 5 out of 32 animals died in the immediate post-operative period resulting in a 16% acute mortality rate. There was no difference in apnea time between scramble-treated (32.3 ± 3.2 s) and AF16-treated animals (33.9 ± 3.2 s). The brain injury induced a peak 10% weight loss at 4 days post-injury (Figure 1B). At 15 days post-injury, the brain-injured animals had significantly lower weight than sham-injured and naïve animals (*p* < 0.05, Figure 1B), not influenced by the treatment compounds.

### Short-term Study—48 h Survival

The primary endpoints in the short-term study using a 48-h post-injury survival were BWC and memory retention in the MWM.

#### Brain Water Content

**Figure 2:**
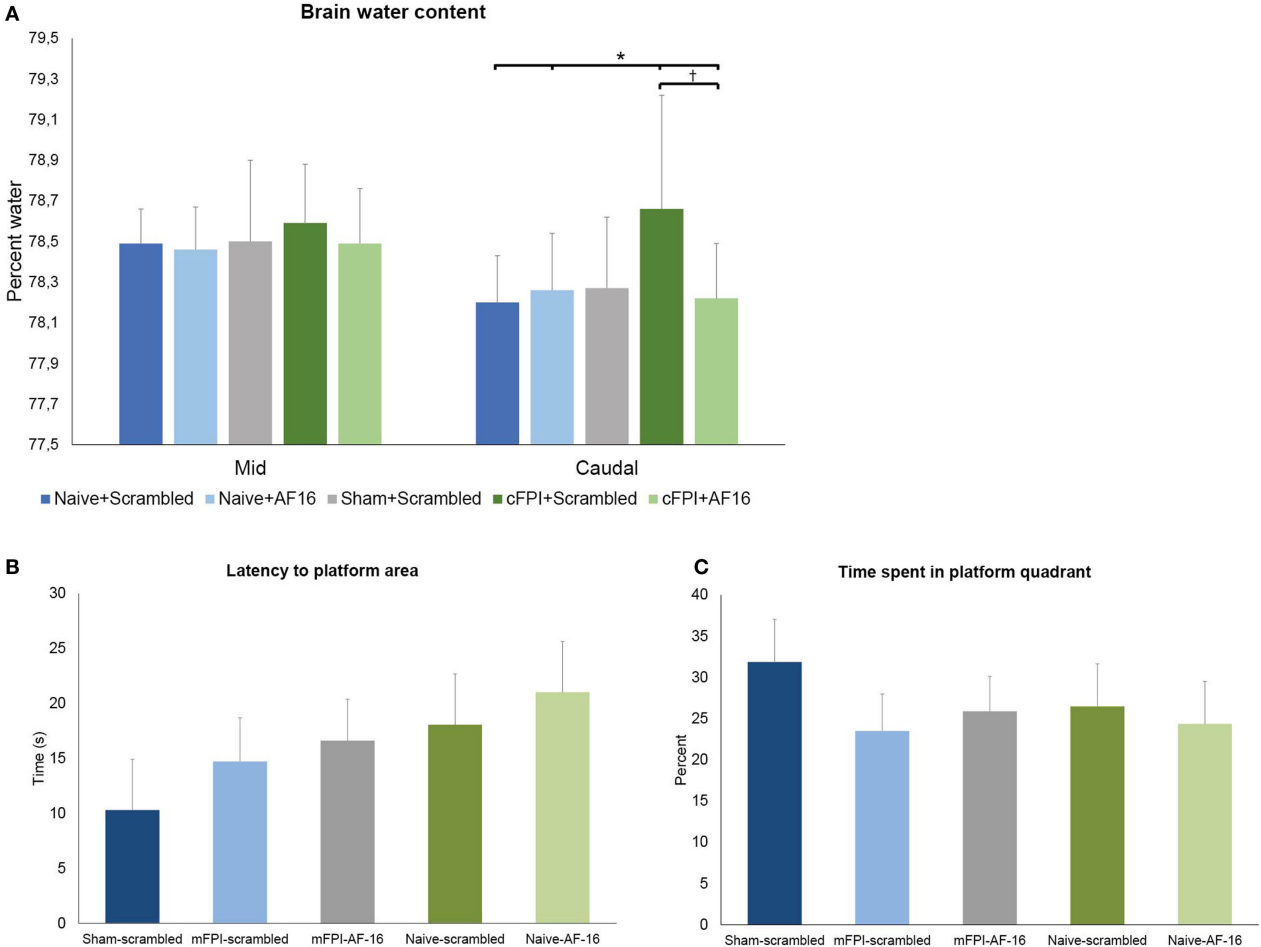
**(A)** Brain water content (BWC) (means ± SEM) at 48 h post-injury in naïve animals, sham-injured controls and animals subjected to the midline fluid percussion brain injury (mFPI) treated with active compound [antisecretory factor (AF)-16] or inactive control substance (scramble). The brain regions underlying the impact were split into two halves. In the caudal brain regions, the mFPI induced a significant (*) increase in BWC compared to all other groups, except the sham-injured scramble-treated group (*p* = 0.11). Treatment with AF-16 following mFPI attenuated the increase in BWC compared to brain-injured, scramble-treated group (#*p* < 0.05). **(B,C)** Memory (probe) trial performed 48 h after sham- or midline fluid percussion injury (mFPI) and in naïve controls. Graphs showing the results from the first 30 s of the memory test in the short-term experiments (means ± SEM). The animals were treated with intranasal administration of either active compound (AF-16) or inactive control substance (Scramble). The latency to locate the platform area did not differ among the different treatment groups **(B)**, neither did the time spent in the platform quadrant **(C)**.

The diffuse brain injury model (mFPI) used in the present report altered the BWC modestly after 48 h when compared to naïve or sham-injured animals (Figure 2). The differences in BWC between groups were small in the two samples obtained from beneath the impact site. In the caudal region, the animals subjected to TBI and treated with scramble peptide had significantly (*p* < 0.05; Figure 2B) higher BWC (78.62 ± 0.56%) compared to naïve scramble-treated animals (78.22 ± 0.23%), naïve AF-16-treated animals (78.31 ± 0.22%), and AF16-treated animals subjected to mFPI (78.27 ± 0.27%), but not sham-injured scramble-treated animals (78.37 ± 0.35%). Thus, treatment with AF-16 attenuated the increased BWC in brain-injured animals.

#### Memory Test in MWM

The animals were trained in the MWM for 2 days prior to the injury. On the second day of pre-training the mean latency to find the platform was 22.6 ± 1.1 s over all four trials. The animals were then randomized in order to get balanced treatment groups.

**Figure 3:**
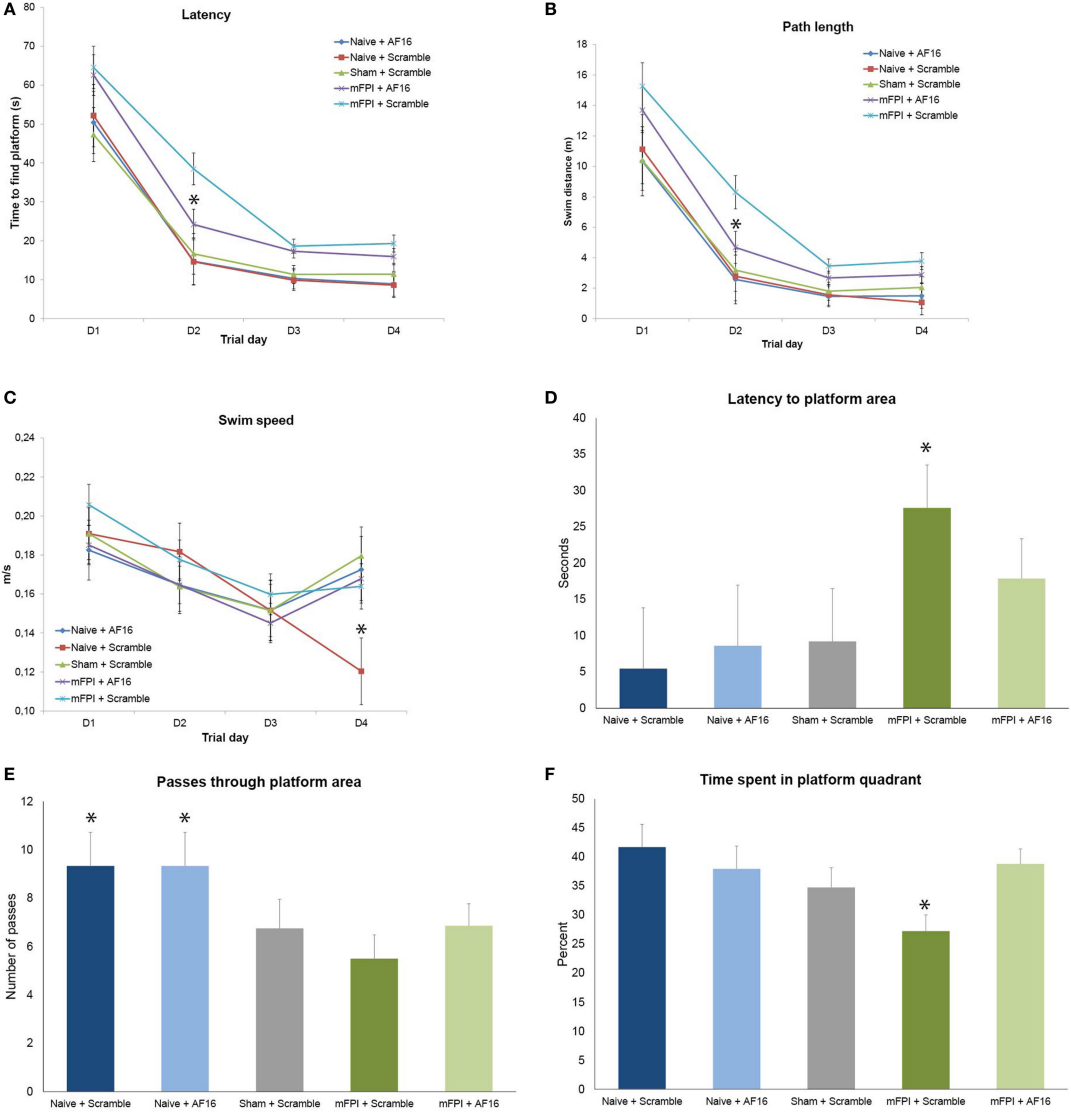
**Results from the Morris Water Maze learning trials (A–C) and memory (probe) trial (D–F) for the 15-day survival study (means ± SEM)**. Rats were subjected to sham- or midline fluid percussion injury (mFPI), and naïve controls were used. The animals were treated with either antisecretory factor (AF)-16 as the active, treatment compound or an inactive control compound (scramble). There was a clear effect on both latency **(A)** and path length **(B)** by the brain injury, without influencing the swim speed **(C)** with the exception of sham-injured, scramble-treated animals that swam significantly slower than all other treatment groups on the fourth day of the trials [*p* < 0.05, **(C)**]. AF-16 treatment significantly decreased the latency to find the platform and path length on the second day of the learning trials compared to scramble-treated, brain-injured controls [*p* < 0.05, **(A,B)**]. The memory (probe) trial at 72 h following the last learning trial showed that scramble-treated, brain-injured animals had significantly longer latency to find the platform area than the naïve and sham-injured treatment groups [*p* < 0.05, **(D)**]. Both naïve treatment groups had significantly more passes through the platform area than sham operated or injured groups [*p* < 0.05, **(E)**]. Scramble-treated, brain-injured animals spent significantly less time in the correct quadrant compared to all other groups [*p* < 0.05, **(F)**]. Treatment with AF-16 in brain-injured animals did not significantly reduce the latency to reach the area originally harboring the hidden platform [*p* = 0.23, **(D)**], increase the passes **(E)**, or increase the time spent in the platform area **(F)**.

The brain-injured animals swam longer and faster than the sham- and uninjured controls (*p* < 0.05; data not shown), though no difference in latency to find the platform area or time spent in the correct quadrant were found (Figures 3A,B). AF16 did not alter the performance in the MWM probe trial compared to scramble treatment in either naïve or brain-injured animals.

### Long-term Study, 9–15 Day Survival

#### Learning Trials at Post-Injury Day 9–12 in the MWM

Rats subjected to mFPI had significantly longer latency (Figure 3A) to find the platform and longer swim distance (Figure 3B) compared to sham-injured and naive animals in all 4 trial days. No differences were found in swim speed between any of the treatment groups (Figure 3C).

Antisecretory factor-16 treatment did not influence any of the MWM parameters in uninjured (sham + naïve) animals (Figures 3A–C). AF-16-treated, brain-injured rats had a shorter latency to locate the hidden platform (*p* < 0.05; Figure 3A) and a shorter swim distance (*p* < 0.05; Figure 3B) on day 2 of the MWM learning trials than scramble-treated, brain-injured rats.

#### Memory (Probe) Trial Test in the MWM, Day 15 Post-Injury

Rats subjected to mFPI had significantly longer latency to enter the platform area compared to both sham- injured and naive animals (*p* < 0.05; Figure 3D). Naïve animals swam over the area originally harboring the platform significantly more times than both brain-injured and sham-injured animals (*p* < 0.05; Figure 3E).

AF-16 treatment did not influence the results of the probe trial in uninjured animals. In brain-injured rats, AF-16-treated had a similar latency to locate the platform area when compared to the scramble-treated, brain-injured rats (*p* = 0.23; Figure 3D). Scramble-treated, brain-injured animals spent significantly less times in the correct platform quadrant when compared to the uninjured groups (*p* < 0.05; Figure 3F). Brain-injured animals treated with AF-16 had more time in the correct platform area compared to brain-injured, scramble-treated animals (*p* < 0.05; Figure 3F).

#### Brain Tissue and Ventricular Volume

**Figure 4:**
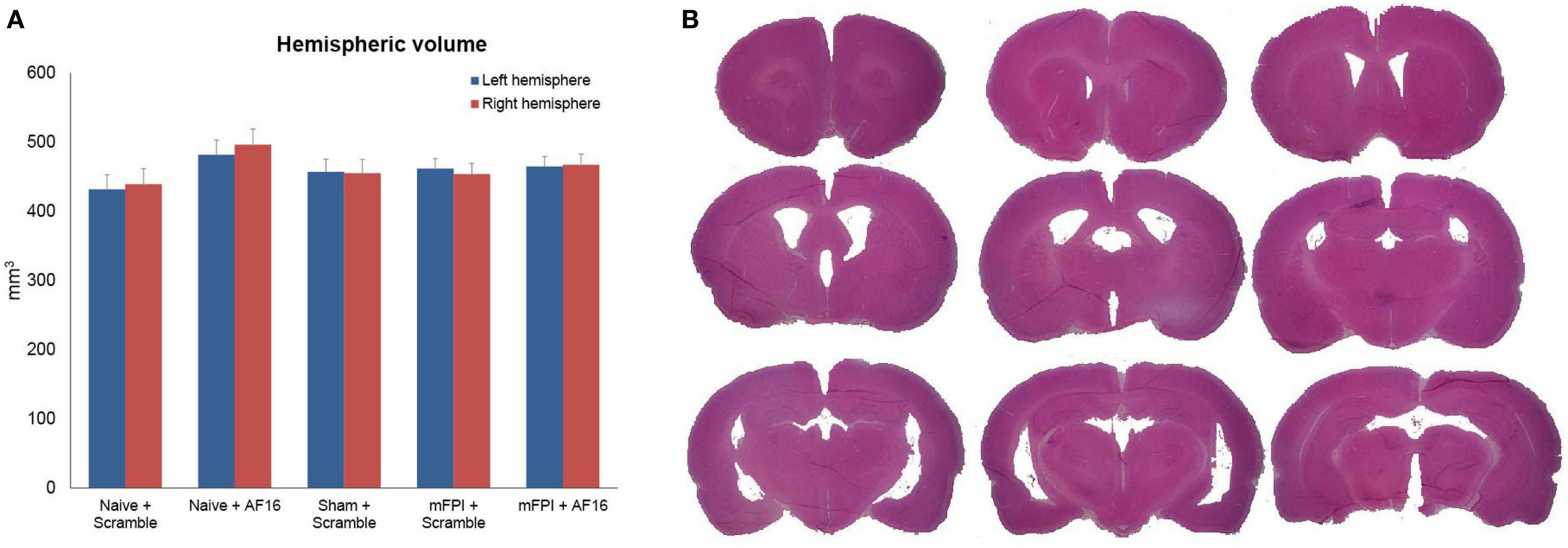
**Hemispheric volumes for left and right hemispheres, showing no statistically significant differences between any of the treatment groups (A)**. Figure **(B)** shows an example of the technique used to estimate tissue volume, using nine sections from a mouse subjected to midline fluid percussion injury (mFPI) and treated with the inactive compound (scramble).

The volumetric analyses did not show any significant difference in brain tissue or lateral ventricular volume at 15 days between any of the treatment groups (Figures 4A,B).

#### Immunohistochemistry

**Figure 5:**
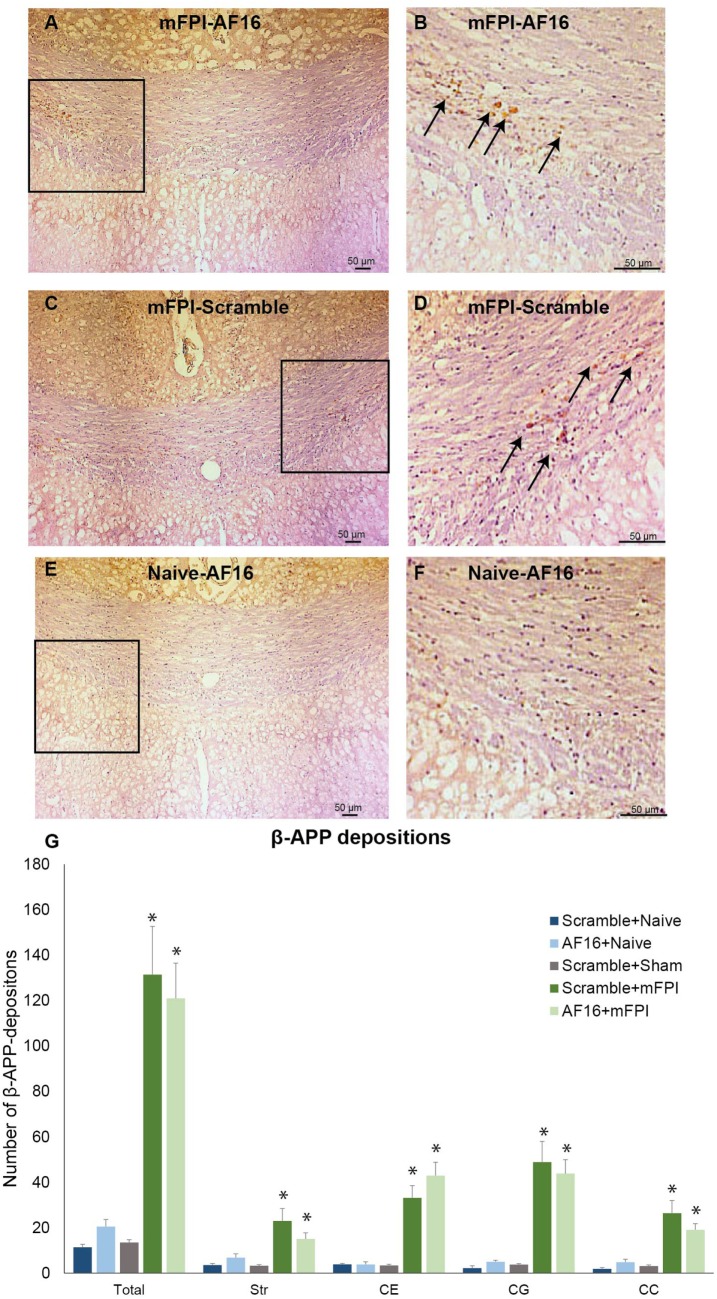
**Immunohistochemistry for beta-amyloid precursor protein (β-APP), a marker for axonal injury, in the corpus callosum at 15 days post-injury**. In left panel **(A,C,E)**, 200× magnification images of animals subjected to midline fluid percussion injury (mFPI, A and C) compared to naïve controls **(E)** are shown. In the right panel **(B,D,F)**, images using 200× magnification are shown. **(A–F)** β-APP deposits indicated by arrows. **(G)** In all regions of interest, a marked injury effect was found (*p < 0.05) although the number of β-APP deposits was not influenced by treatment with antisecretory factor-16 or scramble treatment. Str, striatum; CE, external capsule; CC, corpus callosum; CG, cingulate gyrus.

Compared to sham- and uninjured controls, brain injury using the mFPI model resulted in an increased number β-APP depositions in the white matter (Figure 5), which were not influenced by treatment with either AF-16 or scramble peptide (Figure 5G).

**Figure 6:**
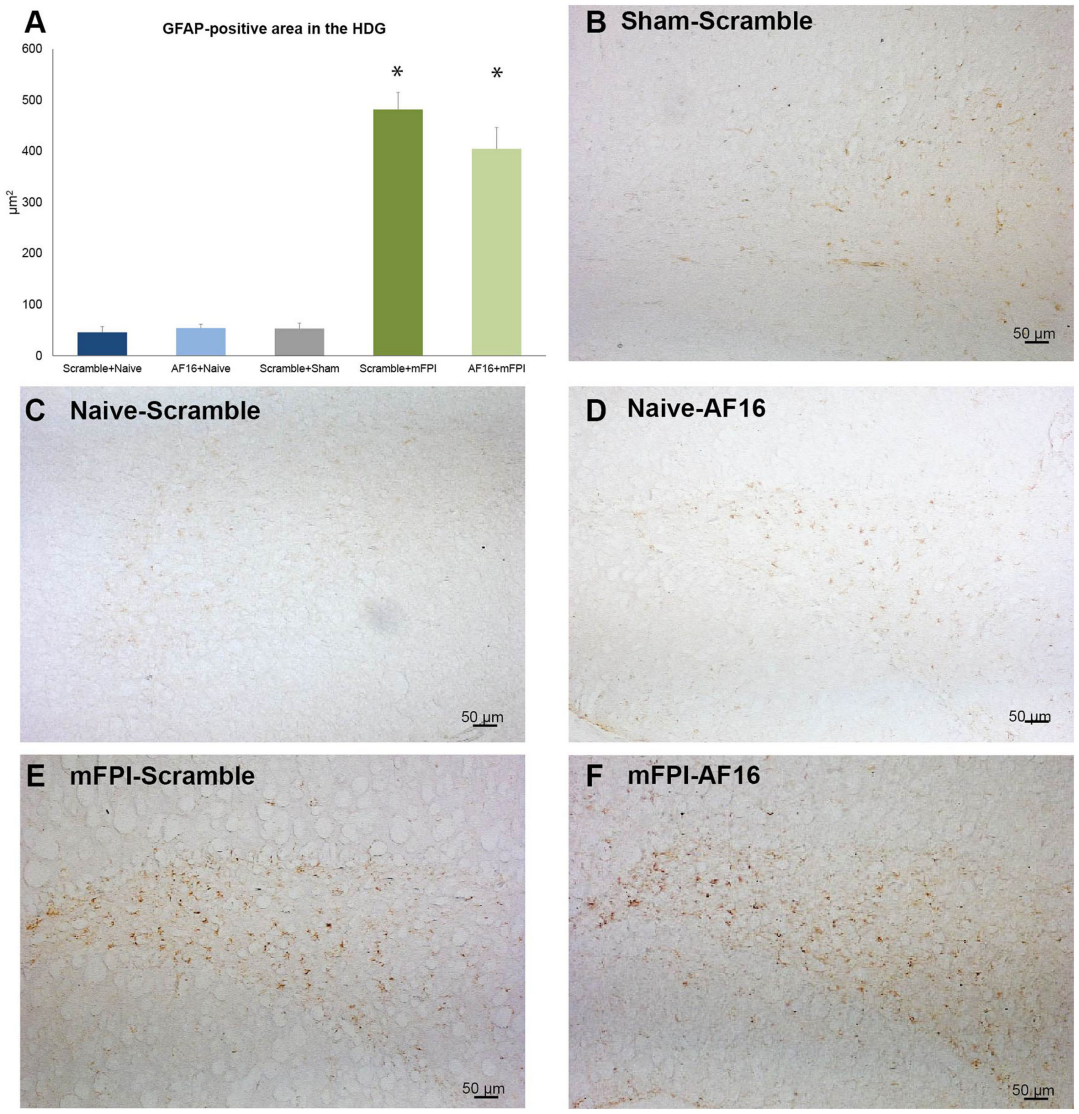
**Immunohistochemistry for glial fibrillary acidic protein (GFAP), an astrocytic marker of the glial scar, in the hilus of the dentate gyrus of the hippocampus at 15 days post-injury**. **(A)** The area of GFAP immunostaining was larger in brain-injured animals (*p* < 0.05). Although the area stained with GFAP was smaller in AF-16-treated, brain-injured animals compared to that of brain-injured, control (scramble)-treated animals it did not reach statistical significance (*p* = 0.107). **(B–F)** Representative examples from sham-injured **(B)**, naïve **(C–D)**, and animals subjected to midline fluid percussion injury (mFPI).

GFAP was used to analyze the HDG region of the hippocampus, where brain injury resulted in an increased GFAP staining compared to the sham- and uninjured animals (*p* < 0.05; Figure 6). Treatment with AF16 showed less GFAP staining although without reaching statistical significance (*p* = 0.11; Figure 5A) compared to scramble-treated, brain-injured animals.

**Figure 7:**
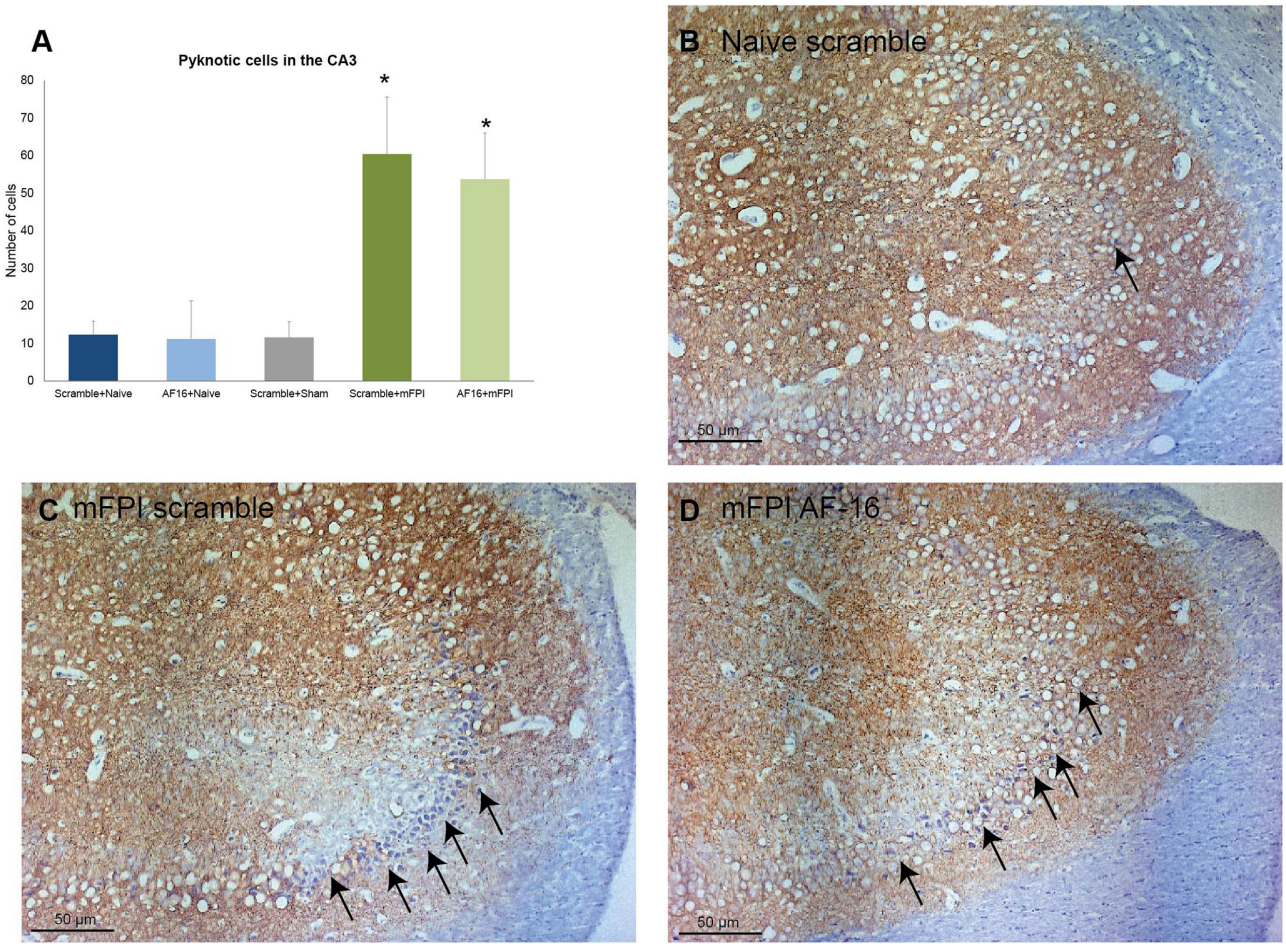
Immunohistochemistry evaluating the number of (A) pyknotic nuclei in the CA3 of the hippocampus at 15 days post-injury, showing an increased number of pyknotic cells in brain-injured animals compared to sham- and naïve controls (*p < 0.05, Figure 8). AF-16-treated animals had similar cell counts as scramble-treated controls, regardless of injury status (Figure 8). **(B–D)** Representative examples from a naïve animals **(B)** and brain-injured animals **(C,D)** subjected to midline fluid percussion injury (mFPI) and treated with inactive control, scramble, compound **(C)**, or the active compound AF-16 **(D)**.

Using MAP2 staining, we measured the unstained area and counted the number of pyknotic nuclei in the CA3 region of the hippocampus. Compared to sham- and uninjured controls, the brain injury resulted in an increased area without MAP2 staining which was not influenced by AF-16 treatment (data not shown). Brain injury also resulted in an increased number of pyknotic cells which was not influenced by AF-16 or scramble treatment (Figure 7).

**Figure 8:**
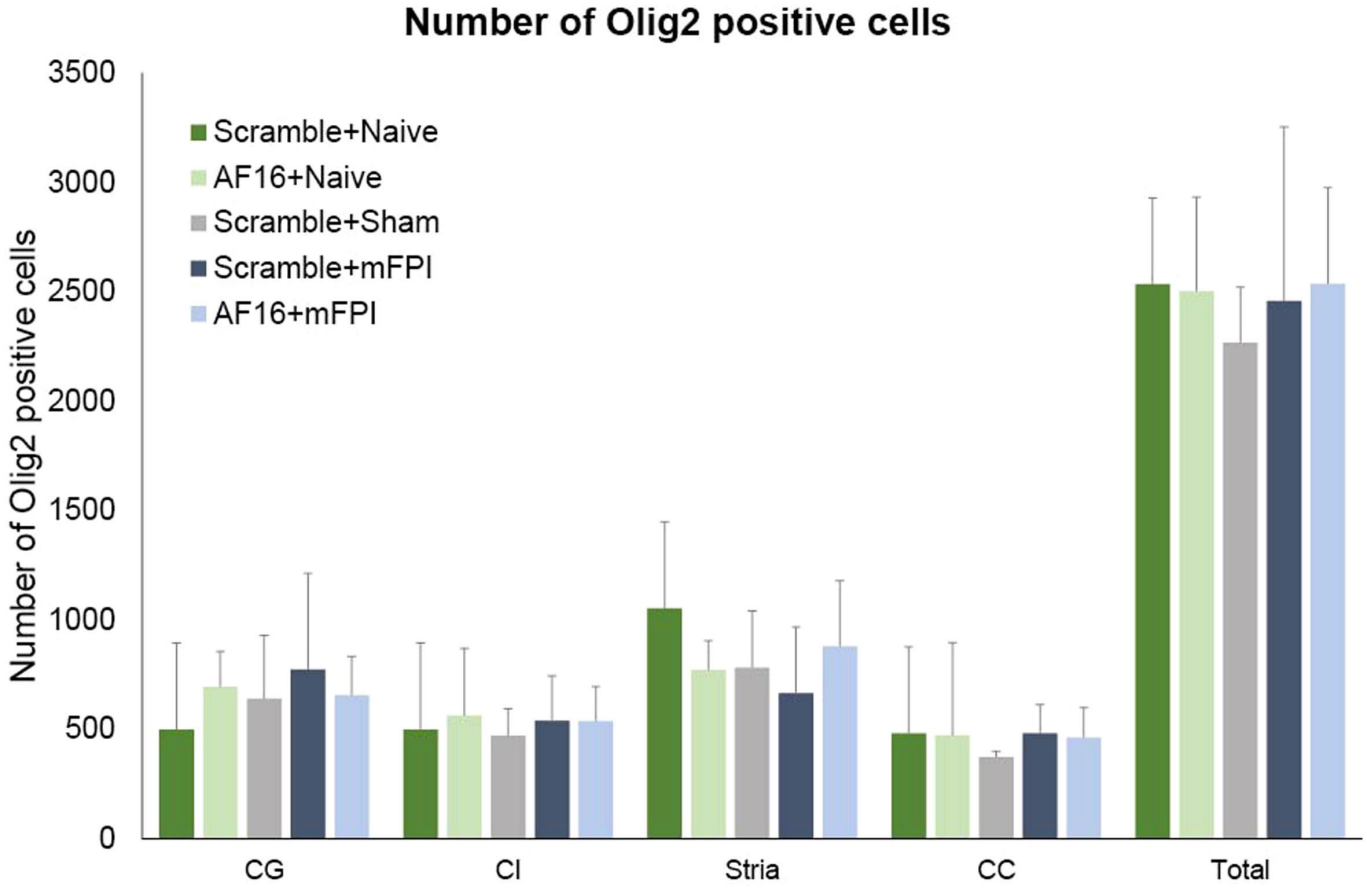
**The number of Olig2-positive cells at 15 days post-injury in selected brain regions of interest following traumatic brain injury using the midline fluid percussion injury (mFPI) model**. The number of Olig2-positive cells were not altered by the injury or by treatment with active (AF-16) or inactive (scramble) compound. Str, striatum; CE, external capsule; CC, corpus callosum; CG, cingulate gyrus.

The immunostaining for Olig2 was used to assess oligodendrocyte and their progenitor cells in selected white matter tracts at 15 days post-injury. No difference was found in the number of Olig2-positive cells between the treatment groups in any of the areas analyzed (Figure 8).

## Discussion

The present study provides evidence that intranasal administration of the antisecretory peptide AF-16 attenuates the TBI-induced brain edema compared to an inactive reference scramble peptide in brain-injured rats at 48 h post-injury. In addition, in the 15-days survival animals the latency to find the hidden platform in the MWM test during the learning trials was shortened by AF-16 treatment, indicating attenuated cognitive deficits by AF-16 administration post-TBI. However, there were no significant effects by AF-16 on brain tissue volume or on the memory tests and no immunohistochemical correlates to the observed improvements in edema and learning abilities were observed. Our results suggest that AF-16 exerts additional pharmacological actions than merely the edema-reducing capacity in order to explain the observed improvements in cognitive function. Such additional mechanisms may include, e.g., immunoregulatory functions of the compound (17).

In the present study, we used the intranasal route for the delivery of the peptide AF-16 aiming to gain access to the brain parenchyma and CSF. Pathways for the delivery of drugs, such as peptides and proteins to the brain from the blood are efficiently restricted by the blood–brain barrier (28). However, several compounds have been found to bypass the BBB using the olfactory nerve. High molecular weight proteins, such as horseradish peroxidase, and peptides, such as insulin-like growth factor I (IGF-I) and insulin, have been conclusively observed to be taken up after nasal instillation, transported to the olfactory bulb and then further throughout the brain including into the CSF (29–32). The peptide AF-16 used in the present report reaches high concentration in the CSF after intranasal instillation (16) and the beneficial effects imply that AF-16 reached the brain parenchyma and retained its biological activity after the intranasal instillation also in the present study. These results are in agreement with those achieved in previous studies where intranasal administration of AF-16 attenuated the rise in ICP and reduced the high mortality in herpes simplex type I encephalitis in rats (18). Further, the elevated ICP developing after a cryogenic brain lesion to rats was lowered by intranasal AF-16 (15, 16). The beneficial effects achieved by intranasal AF-16 administration in the present study argue that the study drug enters the injured brain after mFPI. An advantage of the nasal administration for AF-16 administration is that it is non-invasive, acts rapidly and can be given to almost every TBI patient, even at prehospital situation as well as in children (33–35).

We used MWM performance (short-term study) and apnea time (long-term study) to enable us to get equal experimental brain-injured groups in terms of mFPI impact. In the experimental setting, a transient TBI-induced weight loss is common and reflects injury severity (23, 36). Thus, the midline FPI (mFPI) model of diffuse TBI model used in the present report resulted in a decreased body weight in the first post-injury phase which was not influenced by AF-16 treatment. The mFPI model resulted in a 15% mortality and a 35-s apnea time, consistent with a mild–moderate mFPI (37, 38). The mFPI model results in wide-spread axonal injury as well as neuronal loss in, e.g., the hippocampus and thalamus (39–41). In our hands, the mFPI used in the present study caused limited structural injuries and no hemorrhages (23), again consistent with a mild–moderate TBI. However, edema formation has previously been less exhaustively characterized in this TBI model. In early reports using magnetic resonance imaging 2 and 90 days after mFPI in rats, minor edema in the corpus callosum was observed at 2 days after injury (42). In addition, the vascular reactivity reached the highest level at 24 h after injury (43), suggesting an acute effect of the trauma on the gross morphology of the vascular system of the brain in this TBI model. Here, the mFPI model resulted in an increase in BWC which was significantly attenuated by AF-16 treatment. This post-traumatic increase in BWC elicited by mFPI was modest compared to more focal TBI models such as the lateral FPI model where the BWC increased from 75 to 82% water (44), similar to the BWC increase in the controlled cortical impact model (45, 46).

In previous experimental studies on rats, AF-16 significantly reduced the brain edema after a cold lesion, as determined by the counteraction of the lesion-induced elevation of ICP (15, 16). Increased ICP in experimental diffuse TBI models may lead to neuronal impairment and neuronal death (47). Although the role for increased BWC in axonal injury has not been firmly established, increased ICP is observed in a subset of patients with severe diffuse injury (48, 49). Thus, the attenuation of post-traumatic increase in BWC observed in the present report is likely beneficial.

One of the most devastating consequences of clinical TBI, regardless of severity, is cognitive impairment. Thus, attenuation of cognitive impairment is a key target in the search for novel pharmaceutical targets in experimental TBI research. We observed no effect on memory function by AF-16 at 48 h post-injury; in contrast, the TBI-induced learning deficits were attenuated and memory was improved by AF-16 treatment in the 15-day survival groups. The swim speed data argue that the prolonged latencies to the hidden platform in the MWM are not caused by TBI-induced motor deficits. Additional behavioral tests (50) are needed to establish any effects of AF-16 on more complex fine motor tasks and/or balance function.

The link between post-traumatic brain edema and functional outcome has not been firmly established, but the present and previously published results suggest a beneficial effect by decreasing edema on cognition. Post-injury treatment of rats with the sulphoraphane, a plant substance shown to decrease cerebral edema, improved the performance in the MWM on both learning and memory (51). After TBI in juvenile rats, reduction of edema using siRNA for aquaporin 4 resulted in an improved outcome on learning and neuromotor function (52). In a weight drop model of diffuse TBI in mice, a robust treatment effect on BWC and a corresponding improvement in neuromotor function by minocycline was found (53). In contrast, edema-reducing effects of estrogen sulfate and glibenclamide did not translate into improved functional outcome (54, 55).

Since the improved MWM performance up to 15-days post-injury may not be explained solely by the edema-reducing functions of AF-16 at 48 h post-injury, we then searched for other potential mechanisms using histology.

There was no loss of brain tissue at 15-days after mFPI, confirming our findings from previous work in mice (19) and in rats (56). Although severe diffuse clinical TBI produces a progressive atrophy of the injured white matter tracts (57), it is plausible that at the injury severity used in the present study caused insufficient injury to produce a significant loss of brain tissue in the 2-week observation period. Instead, we searched for immunohistochemical markers that could correlate to the observed cognitive improvement. The immunohistochemical markers were chosen to investigate axonal injury [β-APP (19, 58)], white matter oligodendrocytes [Olig2 (23)] as well as hippocampal astrogliosis [GFAP (59, 60)] and injury to neuronal cells and dendrites [MAP2 (61, 62)].

We observed a markedly increased number of bilateral β-APP deposits in white matter tracts post-injury. While the mFPI resulted in a bilateral increase in deposits β-APP, the numbers were similar in the AF-16 and scramble-treated groups. In the evaluated hippocampi, there was reactive astrogliosis in the dentate gyrus of the hippocampus after injury as well as an increased number of pyknotic cell nuclei and loss of MAP2 staining in the hippocampal CA3 region. Persistent astrogliosis was observed by others following mFPI in the dentate gyrus and CA1 areas of the hippocampus (63), although no difference in the number of astrocytes was observed in another study (64). Here, the hippocampal astrogliosis was not significantly attenuated by AF-16 treatment in brain-injured animals (*p* = 0.11), although we suggest that the observed trend is sufficiently interesting for evaluation at additional post-injury time-points in future studies. Neuronal cell loss in the CA3 of the hippocampus was found to correlate to MWM deficits following mFPI (65), a finding not confirmed in our study. In contrast to our previous study using a shorter survival time-point (23), we did not observe a TBI-induced change in Olig2-positive cells which may be related to the evaluated time point and the injury severity in this study. In the present study, we focused on brain edema, cognitive evaluation, and histological markers. The alterations induced by the injury and/or AF-16 treatment could in additional studies also focus on markers of brain edema such as MMP-9 (66) and neuroinflammation (67) using additional animal groups and time points.

We conclude that we could not identify a single causative histopathological outcome measures explaining the improved functional outcome by AF-16 treatment. It is plausible that the mechanisms are multifactorial although our data may imply that attenuation of hippocampal astrogliosis, plausibly to a higher degree than the effects on the white matter pathology, may be a contributing mechanism.

At the chosen injury severity of the mFPI model, there were no hemorrhages, marked destruction of brain parenchyma, or profound behavioral defects. In clinical practice, most sequelae of mild TBI or brain concussion are associated with white matter pathology including axonal injuries (68). In this TBI model, injury severity needs to be markedly increased to increase BWC. However, one important limitation with the mFPI model is that animal mortality increases substantially when injury severity is increased. Since BWC was only one of several read-outs in this study, our findings point to other mechanisms of action for AF-16. To study the effect of AF-16 on BWC in TBI, other injury models such as the lateral FPI could be used in future studies, since brain edema is more extensive in this injury model (55, 69).

In the present study, only the 2 mg/kg concentration of AF-16 was used which was instilled intranasally twice daily for 2 or 5 days, depending on scheduled post-traumatic survival time. Although a lower AF-16 dose was previously used (18, 24), we chose the 2 mg/kg dose based on effective ICP reductions in cryogenic brain injury with this dosing regimen (16). A recent report indicates that maximal concentrations in the CSF is achieved after about 30 min, and thereafter declines (16). Further studies are required to map the optimal dose and administration regimes required to achieve the optimal treatment effects and brain concentrations. It is important to stress that no adverse side effects by AF-16 were noticed by the selected dose and administration route used here. We also used the MWM as our cognitive test and additional tests, evaluating more complex behavioral changes (19), were not included. To establish a definitive role for AF-16 in TBI, this treatment needs evaluation using additional time windows, focal TBI models, and extended behavioral testing. The promising effects observed in the present study using a clinically relevant route of administration in a diffuse brain injury model warrants further study of AF-16 as a treatment option for TBI.

## Conclusion

We used an experimental model system of diffuse TBI, midline fluid percussion injury (mFPI), in rats to investigate if intranasal administration of a synthetic peptide with antisecretory activity, named AF-16, had any neuroprotective effects. A scrambled, inactive reference peptide was used as a control. At 48 h after mTBI the induced brain edema was significantly counteracted by intranasal instillation of the peptide AF-16 without altering the performance on the memory test in the MWM. In contrast, after 15 days post-injury treatment using AF-16 significantly attenuated the learning deficits in the MWM. No neuroprotective effects could, however, be demonstrated on the histological outcome measures by AF-16 treatment. We conclude that post-injury intranasal administration of the peptide AF-16 attenuates early post-traumatic brain edema and attenuates the cognitive deficits observed at 2 weeks following diffuse TBI in rats. The study of this clinically relevant route of administration of AF-16 is warranted also in other TBI models.

## Ethics Statement

The animal experiments were approved by the Uppsala County Animal Research Ethical Committee application no: C348/11.

## Author Contributions

The experimental procedures were done by FC and H-AH. The data were processed and analyzed by FC and NM. The manuscript was prepared by all authors.

## Conflict of Interest Statement

JR is employed by Lantmännen AS-Faktor AB and H-AH has patents and patent applications related to the AF peptides. FC and NM have no conflicts of interest.
